# The Role of Immunoresolvents in Atherosclerosis: A Mini‐Review on Their Biology and Therapeutic Opportunities

**DOI:** 10.1002/hsr2.73012

**Published:** 2026-08-09

**Authors:** Ramin Lotfi, Alireza Nasirifar, Hoda Rahmani, Sara Falahi, Meysam Kashiri

**Affiliations:** ^1^ Blood Transfusion Research Center High Institute for Research and Education in Transfusion Medicine Tehran Iran; ^2^ Kurdistan Regional Blood Transfusion Center Sanandaj Iran; ^3^ Clinical Research Development Center, Tohid Hospital Kurdistan University of Medical Sciences Sanandaj Iran; ^4^ Lung Diseases and Allergy Research Center, Research Institute for Health Development Kurdistan University of Medical Sciences Sanandaj Iran; ^5^ Department of Internal Medicine School of Medicine, Kurdistan University of Medical Sciences Sanandaj Iran; ^6^ Department of Immunology, Faculty of Medicine Isfahan University of Medical Sciences Isfahan Iran

**Keywords:** atherosclerosis, chronic inflammation, leukotriene B4, resolvin E1, specialized pro‐resolving mediators

## Abstract

**Background and Aims:**

Atherosclerosis is a lipid‐induced chronic inflammatory cardiovascular disorder denoted by the infiltration of immune cells and the agglomeration of lipids in the intima, the innermost layer, of the arteries of large and medium sizes. Defective efferocytosis is a sign of insufficient inflammation resolution that results in the agglomeration of secondarily necrotic macrophages and foam cells, and constitutes an advanced lesion having a necrotic lipid core, indicating plaque vulnerability. The inflammation resolution is an active, highly coordinated process governed by specialized pro‐resolving lipid mediators (SPMs) or immunoresolvents, a group of bioactive lipid mediators that encompass arachidonic acid‐derived lipoxins, omega‐3 fatty acid eicosapentaenoic acid‐derived E‐series resolvins, docosahexaenoic acid‐derived D‐series resolvins, protectins, and maresins. These endogenously produced lipid mediators are resolution ligands functioning on distinct G‐protein coupled receptors, thereby exhibiting the anti‐inflammatory and pro‐resolving effects, restoring tissue homeostasis and healing. Given the involvement of lipids and their pro‐ and anti‐inflammatory derivatives in the pathogenesis of inflammatory states such as atherosclerosis, in this mini‐review, we have tried to give a glance at the role of immunoresolvents in illness pathophysiology and the potential for pro‐resolving therapeutics in atherosclerosis.

**Methods:**

This mini‐review utilized published papers available in electronic databases, including PubMed, Science Direct, and Google Scholar, to recognize pertinent sources through the following search key terms: “Atherosclerosis,” “SPM,” “Immunoresolvent,” “Lipoxin,” “Resolvin,” “Protectin,” and “Maresin” until February 2026.

**Results:**

Recent investigations have shown that imbalances between pro‐inflammatory lipid mediators and immunoresolvents are intimately linked to failed resolution and several common human disorders, including atherosclerosis. Moreover, dysregulation of resolving inflammation in atherosclerosis and other inflammatory diseases has been delineated in recent decades.

**Conclusion:**

Taken together, immunoresolvents could be regarded as new and hopeful therapeutic agents to be studied and utilized for treating atherosclerosis.

**Abbreviations**
AA: arachidonic acid
ASA: acetylsalicylic acid
ATLs: aspirin‐triggered lipoxins
BLT‐1: leukotrien B4 receptor type 1
ChemR23: chemerin receptor 23
CysLT1R: cysteinyl leukotriene receptor 1
DC: dendritic cell
DHA: docosahexaenoic acid
EPA: eicosapentaenoic acid
GPCR: G‐protein coupled receptor
LOX: lipoxygenase
LTB4: leukotriene B4
LXs: lipoxins
NF‐κB: nuclear factor kappa B
NK: natural killer
PD1: protectin D1
PGs: prostaglandins
PPAR‐γ: peroxisome proliferator‐activated receptor‐γ
RvE1: resolvin E1
SPMs: specialized pro‐resolving lipid mediators

## 1 Introduction

Cardiovascular illnesses, such as atherosclerosis, are one of the major underlying causes of morbidity and mortality; hence, they have become a predominant public health problem around the world [1, 2]. Atherosclerosis represents a lipid‐induced chronic inflammatory cardiovascular illness of the vessel walls. It is pathologically denoted by the infiltration of immune cells and the agglomeration of lipids in the innermost layer, termed intima, of the arterial wall with large and medium sizes, resulting in the formation of atherosclerotic plaques [3, 4]. Recent work has revealed that imbalanced pro‐inflammatory and pro‐resolving lipid mediators are involved in the boosting instability of atherosclerotic plaques [5].

Acute inflammation is a physiological response of the host immune system to protect against noxious stimuli, including pathogens, damaged cells, and toxic compounds. Importantly, the acute inflammation is fundamentally triggered within seconds of the identification of dangerous signals, aiming to eliminate the injurious agents and ultimately restore tissue homeostasis [6, 7]. Notwithstanding the potentially protective role of acute inflammation, cumulative evidence increasingly indicates that persistent and uncontrolled inflammatory responses are the underlying cause for the development of numerous systemic and chronic inflammatory disorders, comprising cardiovascular diseases, periodontal diseases, atherosclerosis, diabetes, neurological diseases, asthma, metabolic syndromes, cancer, as well as autoimmune diseases [7, 8, 9].

Generally, the acute inflammatory response is composed of a series of highly coordinated events involving numerous interactions between various cells and their cellular elements that collaborate to maintain homeostasis. Clinically, this response is characterized by the appearance of cardinal signs like heat, redness, swelling, pain, and loss of function [9, 10]. These signs result from cellular and molecular events that include enhanced blood flow and subsequent vascular permeability, infiltration and accumulation of various immune cells at the involved site, and finally, production of pro‐inflammatory mediators, including cytokines, chemokines, and growth factors [6, 11]. Additionally, the derivatives of omega‐6 fatty acid arachidonic acid (AA), such as prostaglandins (PGs) and leukotrienes (LTs), play a critical role in acute inflammation [12, 13].

The ideal consequence of acute inflammation resolution (also referred to as catabasis) is establishing homeostasis, as well as tissue healing and regeneration. Indeed, catabasis is a biochemically active and dynamic process that is crucial for inhibiting the progression of acute to persistent chronic inflammation. This process is partly governed by a group of endogenously synthesized metabolites called specialized pro‐resolving lipid mediators (SPMs) or immunoresolvents that are enzymatically produced from essential dietary fatty acids, including omega‐6 and omega‐3 [6, 14]. Intriguingly, immunoresolvents exert anti‐inflammatory and pro‐resolving actions without suppressing the immune response [8].

In this mini‐review, we present the recent advances concerning the mechanisms related to defective resolving inflammation in atherosclerosis, the receptor‐dependent bioactions of immunoresolvents, and their potential therapeutic applications.

## 2 Methods

For the accomplishment of this mini‐review article, we investigated several databases, including PubMed, Science Direct, and Google Scholar, to recognize pertinent sources via the following search key terms: “Atherosclerosis,” “SPM,” “Immunoresolvent,” “Lipoxin,” “Resolvin,” “Protectin,” and “Maresin” until February 2026. The present mini‐review did not include any data gathering or analysis, but rather focused on reviewing and comparing the findings existing in several scientific investigations associated with these search terms.

## 3 Immunoresolvents: An Overview of the Biosynthesis and Functions

**Figure 1:**
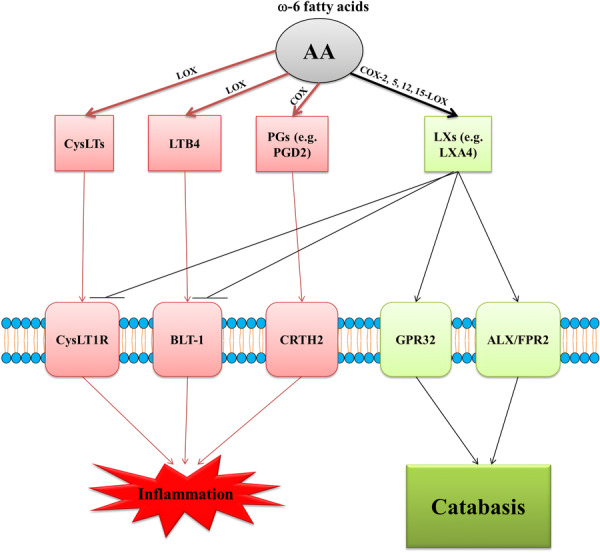
Omega‐6 fatty acids, their derivatives, and receptors in inflammation and resolving inflammation. AA, arachidonic acid; CRTH2, chemoattractant receptor homologous molecule expressed on Th2 cells; CysLT1R, cysteinyl leukotriene receptor 1; LTs, leukotrienes; LX, lipoxin; PGs, prostaglandins.

**Figure 2:**
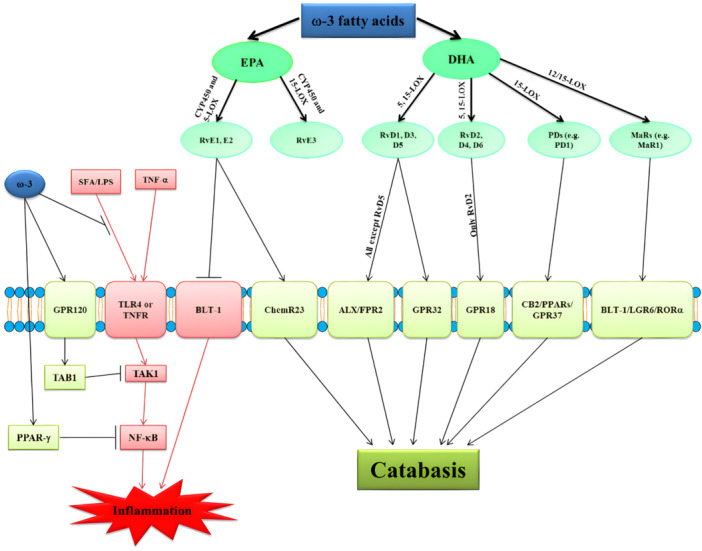
Omega‐3 fatty acids, their derivatives, and receptors in inflammation and resolving inflammation. CB2, cannabinoid receptor‐2; COX, cyclooxygenase‐2; CYP, cytochrome; DHA, docosahexaenoic acid; EPA, eicosapentaenoic acid; LGR6, leucine‐rich repeat‐containing G protein‐coupled receptor 6; LOX, lipoxygenase; MaR, maresin; NF‐κB, nuclear factor kappa B; PPAR‐γ, peroxisome proliferator‐activated receptor‐γ; RORα, retinoic acid‐related orphan receptor α.

Immunoresolvents, including lipoxins (LXs), resolvins (Rvs), protectins (PDs), and maresins (MaRs), are a series of anti‐inflammatory compounds derived primarily from essential fatty acids (i.e., omega‐6 and omega‐3 fatty acids) that serve as pro‐resolving mediators [6]. Indeed, immunoresolvents are produced through lipoxygenase (LOX)‐mediated enzymatic reactions. LXs, the lead members of the immunoresolvents family, are distinct from inflammatory mediators' PGs and LTs in terms of structure and function; however, they are also generated from the metabolization of omega‐6 fatty acid AA (C20:4 ω‐6) (Figure 1). Apart from the AA, the omega‐3 eicosapentaenoic acid (EPA; C20:5 ω‐3) and docosahexaenoic acid (DHA; C22:6 ω‐3) can also be metabolized by LOX‐mediated enzymatic reactions into immunoresolvents, encompassing Rvs, PDs, and MaRs (Figure 2) [10]. Notably, the anti‐inflammatory actions of omega‐3 fatty acids are generally exerted by three major mechanisms: (1) restraining AA conversion into pro‐inflammatory LTs (like 4‐series LTs) and PGs (like 2‐series PGs) via substrate competition; (2) generating less strong inflammatory eicosanoids such as 5‐series LTs and 3‐series PGs, and thromboxanes by functioning as an alternative substrate; (3) producing immunoresolvents (including Rvs, PDs, and MaRs) with anti‐inflammatory and pro‐resolving features through multiple enzymatic reactions on EPA and DHA [6, 15]. Importantly, widespread investigations have depicted that the pro‐resolving functions of immunoresolvents are principally fulfilled via activating one or more G protein‐coupled receptors (GPCRs) [16, 17]. Besides, additional anti‐inflammatory effects of omega‐3 fatty acids or their derivatives include changing the composition of fatty acids in membrane phospholipids, impairment of lipid rafts, and diminishing the gene expression of inflammatory factors by restraining the induction and then activation of a pro‐inflammatory transcription factor called nuclear factor kappa B (NF‐κB) [6, 18]. Figures 1 and 2 illustrate how the anti‐inflammatory immunoresolvents interact with key pro‐inflammatory mediators and their receptors.

## 4 Anti‐Inflammatory Mediators Generated From AA

### 4.1 LXs

LXs are derived enzymatically from the metabolism of omega‐6 AA; thus identifying as the downstream metabolites of this fatty acid. The specific phospholipase A2 enzymes release AA from the sn‐2 fatty acyl bond of membrane phospholipids upon cell activation [19]. The term LX originates from the phrase “lipooxygenase interaction products,” which refers to the LOX‐mediated production of LXs via multicellular interactions [6, 10]. LXs‐ having two members of LXA4 and LXB4, represent the first identified SPMs causing inflammation resolution [20]. They play key roles in resolving inflammation and have anti‐inflammatory properties [21]. LXs have been shown to influence lipid metabolism, which is central to the development of atherosclerosis. They can alter the uptake and efflux of lipoproteins, potentially impacting lipid accumulation in the arterial wall. Unlike many other lipid mediators, which promote inflammation, LXs help restore tissue homeostasis and facilitate the healing process following an inflammatory response. LXs can inhibit the chemotaxis of neutrophils and promote macrophage uptake of apoptotic cells, which is critical for resolving inflammation. They facilitate repair processes by influencing cell migration and proliferation [22]. LXA4, the most studied LX, was the first recognized immunoresolvent among LXs [19].

**Figure 3:**
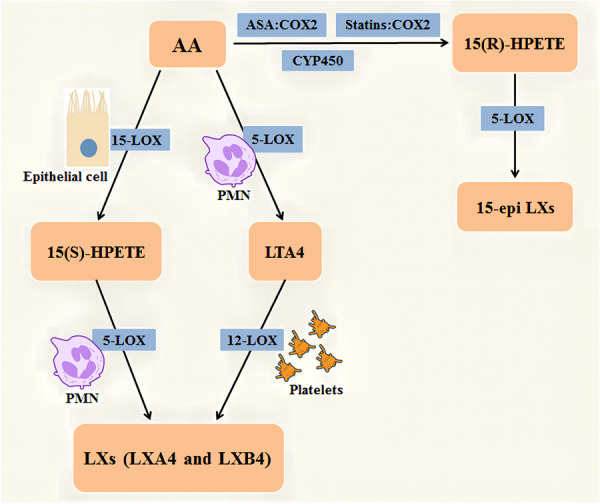
The major biosynthetic pathways of LXs and 15‐epi LXs. LXs and 15‐epi LXs originate from omega‐6 fatty acid AA. When circulating leukocytes enter the lung, their 5‐LOX enzyme can cooperate with airway epithelial cell‐derived 15‐LOX to produce LXs. The biosynthetic intermediates originated from AA through 15‐LOX, like 15(S)‐hydroperoxy‐eicosatetraenoic acid (15(S)‐HpETE), can act as substrates to be converted into LXs by neutrophil‐derived 5‐LOX. Another route includes interactions occurring between platelets and leukocytes. Leukocyte‐derived 5‐LOX turns AA into LTA4. When platelets adhere to neutrophils, their 12‐LOX turns LTA4 into LXA4 and LXB4. Besides that, biosynthesis can take place through the action of airway CYP450, ASA‐acetylated COX‐2, or statin‐S‐nitrosylated COX‐2, resulting in the production of 15‐epi‐LXs during cell‐to‐cell interactions. AA, arachidonic acid; ASA, acetylsalicylic acid; COX, cyclooxygenase; CYP450, cytochrome P450; LOX, lipoxygenase; LT, leukotriene; LX, lipoxin. Adapted from Barnig et al. [10]. https://doi.org/10.1016/j.pharmthera.2018.01.004.

At the sites of inflammation, LXs are locally generated from AA by two major pathways mediated by LOX. These metabolites are biosynthesized and liberated by leukocytes that express 5‐LOX, using the 15(S)‐hydroperoxy‐eicosatetraenoic acid (15(S)‐HPETE) unstable intermediate as a substrate, which is generated in the 15‐LOX‐expressing epithelial cells [10, 23]. Additionally, interactions occurring between platelets and neutrophils can turn AA into LTA4 by the 5‐LOX enzyme, which is expressed in neutrophils. Afterward, LTA4 can be exported into platelets to generate LXA4 and LXB4 by 12‐LOX, which is expressed in platelets [6, 10]. Several therapeutic medicines also stimulate the formation of LX epimers. For instance, aspirin (also known as acetylsalicylic acid [ASA]) is depicted to acetylate and thus inactivate cyclooxygenase‐2 (COX‐2) to restrain the AA‐derived PGs generation, thereby inducing the AA conversion to 15(R)‐HPETE. After that, 15(R)‐HPETE is turned into 15 epimers of LXs (15‐epi LXs) by 5‐LOX action. Since ASA stimulates this route, the produced metabolites from this route (i.e., 15‐epi LXs) are generally called aspirin‐triggered lipoxins (ATLs) [24]. Statins can also induce the 15‐epi LXs biosynthesis in the airway through the COX‐2 S‐nitrosylation [25, 26]. Moreover, hydroxylation of AA, which is mediated by cytochrome P450 (CYP450), is the third way to generate 15(R)‐HPETE and, then, 15‐epi LXs (Figure 3) [27].

LXs and epi‐LXs exert their anti‐inflammatory actions via a 7‐transmembrane GPCR named lipoxin A4 receptor/formyl peptide type 2 receptor (ALX/FPR2; also referred to as formyl peptide receptor‐like 1), which belongs to the N‐formyl peptide receptor family. Engagement of these receptors with LXs and epi‐LXs activates downstream signaling pathways that mediate their anti‐inflammatory actions [28]. Indeed, binding of LXs or epi‐LXs to the ALX/FPR2 triggers the cytoplasmic signaling cascades that attenuate the nuclear agglomeration of inflammatory transcription factors, like NF‐κB and activation protein‐1, which are required for interleukin (IL)‐8 generation, consequently diminishing neutrophil influx and activation [29, 30]. Moreover, LXs have been demonstrated to increase the nuclear levels of the strong anti‐inflammatory receptor named peroxisome proliferator‐activated receptor‐γ (PPAR‐γ) [29, 31]. The receptor of ALX/FPR2, which is expressed by human neutrophils, monocytes, macrophages, eosinophils, T and natural killer (NK) cells, fibroblasts, type 2 innate lymphoid cell (ILC2), and airway epithelial cells, has also been indicated to bind to both bioactive lipid mediators and peptides, for example, resolvin D1 (RvD1) and annexin 1, in order [6, 32]. ALX/FPR2 can either induce or resolve inflammation owing to having over 30 ligands with various affinities [33, 34]. Indeed, ALX/FPR2 is a pleiotropic GPCR characterized by substantial ligand diversity. Importantly, this ligand diversity is associated with functional selectivity (biased agonism), whereby distinct ligands stabilize different receptor conformations and preferentially activate discrete downstream signaling pathways. Pro‐resolving ligands such as LXA4 and RvD1 typically promote Gi‐dependent signaling, inhibition of NF‐κB activation, enhanced efferocytosis, and attenuation of pro‐inflammatory cytokine generation. In contrast, serum amyloid A engagement of ALX/FPR2 has been linked to the extracellular signal‐regulated protein kinases 1 and 2 (ERK1/2) activation, NF‐κB signaling, and increased expression of adhesion molecules. These divergent signaling outputs underscore that ALX/FPR2 activation is not uniformly anti‐inflammatory but is highly context‐ and ligand‐dependent, an issue of central importance for therapeutic translation [35]. Notably, LXA4 has been indicated to antagonize the LTD4 receptor called cysteinyl leukotriene receptor 1 (CysLT1R) (Figure 1). Nonetheless, the receptor for LXB4 has yet to be discovered. Furthermore, LXs bind to additional receptors, comprising GPR32/DRV1 (RvD1 receptor), aryl hydrocarbon receptor (AHR; a ligand‐activated transcription factor), and estrogen receptors [6, 10, 29, 36].

Comprehending the involvement of LXs in resolving inflammation is important for recognizing potential therapeutic targets and mechanisms for managing atherosclerosis and its complications [5, 37]. In 2010, Ho et al.'s study showed that plasma concentrations of ATL are significantly reduced in patients with symptomatic peripheral artery disease compared to healthy controls. Also, this study indicated that ATL blocks platelet‐derived growth factor (PDGF)‐induced migration of human saphenous vein smooth muscle cells and reduces the platelet‐derived growth factor receptor‐β (PDGFR‐β) phosphorylation [1]. In another study examining the effects of ATL and its receptor ALX/FPR2 on the development and advancement of atherosclerosis in apolipoprotein E‐deficient (*ApoE^−/−^*) mice, it was shown that ATL blocks atherosclerosis advancement in the aortic root and thoracic aorta of *ApoE^−/−^* mice. Likewise, ATL decreased the influx of macrophages and apoptotic cells in the atherosclerotic lesions [38]. High levels of plasma LXA4 were also found to be linked with a lower risk of recurrent ischemic events for acute myocardial infarction patients, which could act as a new therapeutic target to tackle cardiovascular inflammation [39]. Recently, the plasma levels of LXA4 and the ratio of LXA4 to pro‐inflammatory IL‐6 cytokine have been reported to display a gradual reduction across control, low‐risk, medium‐risk, and high‐risk groups of coronary heart disease (CHD) and indicated a negative association with the Synergy Between Percutaneous Coronary Intervention with Taxus and Cardiac Surgery (SYNTAX) score, which is a tool for assessing the complexity and risk related to CHD. Analysis of correlations also revealed the negative associations between plasma LXA4 and both inflammatory C‐reactive protein (CRP) and IL‐6 mediators, and a positive relationship with the anti‐inflammatory IL‐10 cytokine. Moreover, the ratio of LXA4 to IL‐6 has been found to be indicative of long‐lasting prognosis [40]. AT‐LXB4 plasma levels were lower in patients with coronary artery disease (CAD), suggesting a striking reduction in resolving capacity for acute inflammation or failed local resolution that could likely result in chronicity or persistent uncontrolled inflammation in these patients [41]. Moreover, imbalanced LXA4 and CysLTs production, favoring persistent airway inflammation and airflow obstruction, has formerly been indicated in individuals with severe asthma [42]. Consistent with this finding, evidence also demonstrated an imbalance in the release of pro‐inflammatory leukotriene B4 (LTB4) and anti‐inflammatory LXA4 lipid mediators from stimulated whole blood of localized aggressive periodontitis patients [43].

Owing to their role in resolving inflammation, LXs and their analogs are being investigated for potential therapeutic applications in various inflammatory diseases, such as asthma, cardiovascular diseases, and autoimmune disorders. Ongoing research is exploring the broader roles of LXs in cellular signaling, their interactions with other lipid mediators, and their potential in personalized medicine. Overall, LXs represent an essential aspect of the body's innate ability to regulate inflammation and contribute to disease resolution [23]. Strategies aimed at enhancing LX signaling or mimicking its action may provide new avenues for preventing and treating atherosclerosis and associated cardiovascular diseases. The potential of LXs to augment the timely resolution of ongoing inflammation and support endothelial function positions them as critical factors in the development and progression of atherosclerotic disease. Continued research into their mechanisms and therapeutic potential could lead to novel interventions for cardiovascular disease management.

## 5 Anti‐Inflammatory Mediators Produced From EPA and DHA

### 5.1 E‐Series Rvs

**Figure 4:**
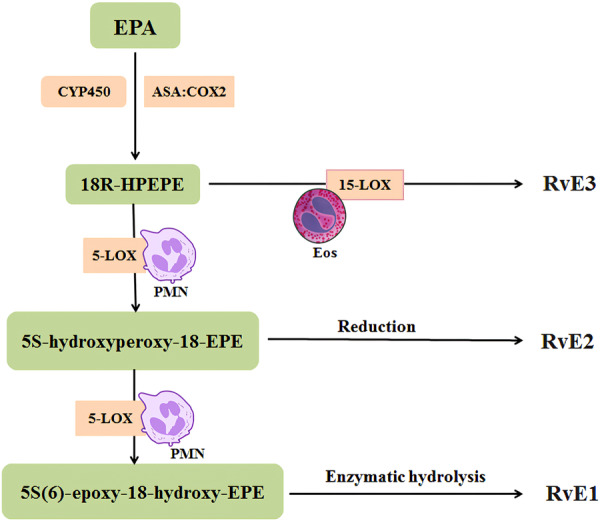
The biosynthetic pathways of EPA conversion into E‐series resolvins. EPA is turned into the 18R‐hydroperoxy‐eicosapentaenoic acid (18R‐HpEPE) intermediate through ASA‐acetylated COX‐2 or using CYP450 in microbes. The 18R‐HpEPE intermediate can undergo further transformation by PMN‐derived 5‐LOX to an intermediate for the next enzymatic conversion to RvE1 or through reduction to RvE2. RvE3 is directly produced from 18R‐HpEPE through 15‐LOX expressed on eosinophils. ASA, acetylsalicylic acid; COX, cyclooxygenase; CYP450, cytochrome P450; Eos, eosinophil; EPA, eicosapentaenoic acid; LOX, lipoxygenase; PMN, polymorphonuclear cell; RvE1, resolvin E1. Adapted from Barnig et al. [10]. https://doi.org/10.1016/j.pharmthera.2018.01.004.

E‐series Rvs (i.e., RvE1 to RvE3; E nomenclature implies the first letter of EPA) are enzymatically produced from EPA by the activity of aspirin‐mediated COX‐2 acetylation (acetyl COX‐2) and 5/15‐LOXs or CYP450 [44, 45]. Utilizing either CYP450 or acetylated COX‐2, EPA is turned into the intermediate 18 R‐HPEPE, which can be transported into eosinophils and afterward turned into RvE3 by using eosinophil‐derived 15‐LOX. Also, 18 R‐HPEPE can be turned into the intermediate 5S‐hydroxyperoxy‐18‐EPE via neutrophil‐derived 5‐LOX. The process of reduction of 5S‐hydroxyperoxy‐18‐EPE generates RvE2. Besides, 5S‐hydroxyperoxy‐18‐EPE can be transported into neutrophils and turned into 5S(6)‐epoxy‐18‐hydroxy‐EPE, which then undergoes enzymatic hydrolysis, thereby producing RvE1 (Figure 4) [10]. Resemble LXs, the aspirin‐triggered immunoresolvents are also generated from both EPA and DHA [46].

RvE1 exerts strong anti‐inflammatory functions by restraining the endothelial transmigration of neutrophils and their responsiveness to inflammatory stimulants via increasing CCR5 expression and inhibiting NF‐κB activation, in order [6]. Also, RvE1 can enhance the clearance of apoptotic neutrophils via macrophage‐mediated phagocytosis and efferocytosis, a non‐phlogistic clearance of cellular debris mediated by phagocytes. Moreover, RvE1 acts to inhibit dendritic cell (DC) migration, cytokine secretion, and the levels of pro‐inflammatory peptide mediators [47]. RvE1 has been shown to prevent DC maturation by the regulation of cytokine secretion from DCs, Th17 subset differentiation from naive T cells, Th17 chemoattraction, and T cell induction and then activation [48]. Generally, the bioactions of RvE1 are fulfilled via at least two distinct receptors which belong to the GPCRs family, comprising chemerin receptor 23/the receptor for the RvE1 (ChemR23/ERV1: also referred to as CMKLR1: chemokine‐like receptor 1) and leukotriene B4 receptor type 1 (BLT‐1), which is also a receptor for the LTB4 pro‐inflammatory mediator. Of interest, RvE1 has been indicated to connect to ChemR23/ERV1 even with higher affinity relative to chemerin, a peptide ligand that is also found to be able to connect to ChemR23 [45, 49]. Indeed, RvE1 functions as an agonist for the ChemR23 receptor, which is frequently found on monocytes, macrophages, NK cells, ILC2, DCs, lung epithelial cells, plasmacytoid DCs, and, with lesser quantities, on T cells and neutrophils [6, 10, 32, 50, 51, 52, 53]. Also, RvE1 can connect to the BLT‐1 receptor, resulting in the inhibition of LTB4‐stimulated recruitment of neutrophils. It has been indicated that BLT‐1 is found on the surface of neutrophils, monocytes, eosinophils, and T cells. Accordingly, the pro‐resolving function of RvE1 can be explained by two main mechanisms: (a) the agonistic function of RvE1 for the anti‐inflammatory ChemR23 and (b) its antagonistic function for the pro‐inflammatory BLT‐1 [6, 36]. RvE1 also blocks PDGF‐induced migratory responses of human saphenous vein smooth muscle cells and diminishes the PDGFR‐β phosphorylation [1].

The research examining the therapeutic effect of RvE1 on the beginning of atherosclerosis in the rabbit model indicated that oral/topical RvE1 considerably decreased atherogenesis and prevented periodontitis. In the absence of periodontitis, oral/topical application of RvE1 led to dramatically less arterial plaque, a lower intima/media ratio, and reduced inflammatory cell influx relative to no treatment. Local oral RvE1 application also markedly diminished systemic CRP levels [54]. In an experimental model of diet‐stimulated atherosclerosis, that is, the ApoE*3Leiden transgenic murine, it was demonstrated that RvE1 attenuates atherosclerotic lesion development when injected orally once daily for 16 weeks in mice with established progressive atherosclerosis. Also, RvE1 decreased total lesion area by 35%, and in animals treated with RvE1, the proportion of atherosclerotic lesions was considerably skewed towards mild (type I) lesions over more severe (type IV and V) lesions [55]. RvE1 plasma levels were reported to be lower in CAD patients, suggesting a considerable reduction in resolving capacity for acute inflammation or failed local resolution that could probably result in chronicity or persistent uncontrolled inflammation in these patients [41]. Recent work in 2023 has indicated that the serum concentrations of LTB4 are dramatically increased in patients with atherosclerosis when compared to those of normal subjects. Also, the serum EPA and RvE1 concentrations were meaningfully greater in these patients than in normal subjects. Nonetheless, the RvE1 to LTB4 ratio (RvE1:LTB4) in these patients was strikingly reduced compared to that in normal subjects. The findings of this study show that an imbalance between pro‐resolving RvE1 and pro‐inflammatory LTB4 might result in failing vascular inflammation resolution and subsequent advancement toward chronic unresolved inflammation in atherosclerosis [4]. Similarly, imbalances between RvE1 and LTB4 that may contribute to defective airway inflammation resolution and subsequent advancement toward chronic inflammation have previously been shown in patients with allergic rhinitis [56].

### 5.2 Metabolites Derived From DHA: D‐Series Rvs, PDs/Neuroprotectins (NPDs), and MaRs

D‐series Rvs (i.e., RvD1 to RvD6; D nomenclature implies the first letter of DHA), NPDs/PDs, and MaRs are formed from DHA by the enzymatic activity of human 15‐LOX and mouse 12/15‐LOX [44, 57].

#### 5.2.1 D‐Series Rvs

**Figure 5:**
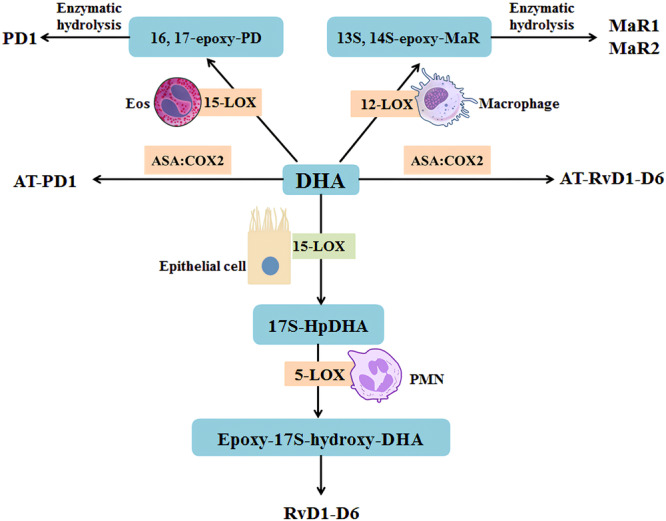
The biosynthetic pathways of DHA conversion into D‐series resolvins, protectins, and maresins. DHA is turned into the 17S‐hydroperoxy‐DHA (17‐HpDHA) intermediate by epithelial cell‐derived 15‐LOX enzymatic action, which subsequently undergoes further conversion by neutrophil‐derived 5‐LOX to epoxide intermediates that are precursors to the D‐series resolvins, that is, RvD1 to RvD6. Through macrophage 12‐LOX, DHA is also turned into a 13S, 14S‐epoxy‐maresin intermediate and, afterward, via enzymatic hydrolysis to MaR family members, including MaR1 and MaR2. Through eosinophil 15‐LOX, DHA can also be turned into a 16, 17‐epoxy‐protectin intermediate, which is turned into PD1/NPD1. Low‐dose ASA causes the activation of the 17R and 18R/S epimers of the resolvins (AT‐Rvs) and 17R‐epimer protectins (AT‐PD1). ASA, acetylsalicylic acid; COX, cyclooxygenase; DHA, docosahexaenoic acid; Eos, eosinophil; LOX, lipoxygenase; MaR, maresin; PD1/NPD1, protectin D1/neuroprotectin D1; PMN, polymorphonuclear cell; RvD1, resolvin D1. Adapted from Barnig et al. [10]. https://doi.org/10.1016/j.pharmthera.2018.01.004.

D‐series Rvs are biosynthesized from the primary metabolism of DHA by using a wide variety of cells and tissues [58, 59, 60]. Using 15‐LOX expressed by airway epithelial cells, DHA can be turned into 17S‐hydroperoxyDHA (17S‐HpDHA), which is then converted into a precursor named epoxy‐17S‐hydroxy‐DHA for the biosynthesis of RvD1‐D6 by neutrophil‐derived 5‐LOX. Besides, DHA can be turned into the epimers of RvDs called AT‐RvD1‐6 via acetyl COX‐2 (Figure 5) [10, 61].

The recognized receptors for RvD1 include ALX/FPR2 and GPR32/DRV1, which is an orphan GPCR [58, 59]. RvD1 has been shown to enhance macrophages' phagocytic functions, but it slows down their chemotaxis toward the CCL2 chemokine. Also, RvD1 could limit the influx of inflammatory monocytes to the sites of inflammation via powerful inhibition of pro‐inflammatory cytokines (such as IL‐1β, IL‐8, and CCL2) generation from human macrophages in a way that is dependent on the GPR32. The expressions of CD206, arginase 1, chitinase‐3‐like protein 3 (YM1), and IL‐10, which are associated with an anti‐inflammatory macrophage phenotype (i.e., M2‐type macrophage), are also enhanced by RvD1 in macrophages related to the inflamed adipose tissue [62].

The levels of RvD1 and the SPMs ratio to pro‐inflammatory LTB4 mediator were found to be markedly diminished in the vulnerable human plaques obtained from carotid endarterectomy. SPMs were also reduced in advanced plaques of fat‐fed *Ldlr^−/−^* mice. Injecting RvD1 into these mice during plaque advancement restored the RvD1:LTB4 ratio to that of less advanced lesions and augmented plaque stability, comprising reduced lesional oxidative stress and necrosis, bettered lesional efferocytosis, and thicker fibrous caps [5]. RvD1, RvD2, RvD3, RvD5, and AT‐RvD3 plasma levels were found to be decreased in CAD patients, suggesting a remarkable reduction in resolving ability for acute inflammation or failed local resolution that could likely result in chronicity or persistent uncontrolled inflammation in these patients [41]. Also, plasma concentrations of RvD1 and the ratio of RvD1 to IL‐6 cytokine have recently been reported to display a gradual reduction across control, low‐risk, medium‐risk, and high‐risk groups of CHD, and illustrated a negative association with the SYNTAX score. Analysis of correlations also unveiled the negative associations between plasma RvD1 and both inflammatory CRP and IL‐6 mediators, and a positive relationship with the anti‐inflammatory IL‐10 cytokine. Moreover, the ratio of RvD1 to IL‐6 has been found to be indicative of long‐lasting prognosis [40]. In *ApoE^−/−^* mice, increased levels of LTB4 were found to be directly correlated with plaque vulnerability, while RvD2 was associated with plaque stability, indicating the imbalance between pro‐inflammatory LTB4 and pro‐resolving RvD2 lipid mediators during atheroprogression [63].

#### 5.2.2 PDs or NPDs

PDs, also referred to as NPDs, are produced from DHA. Amongst PDs, protectin D1 (PD1) is one of the most investigated [6]. Notably, when PD1 is generated in the nervous system, the name NPD1 is utilized to refer to the location of strong protective bioactions in the brain, retina, and pain induction [64]. PDs, resembling other immunoresolvents, thwart activated leukocytes and boost the resolving phase of inflammation [65]. Human eosinophils that express 15‐LOX are able to turn DHA into 16‐, 17‐epoxy‐PD, which can then be turned into PD1 via enzymatic hydrolysis. AT‐PD1 is also produced from DHA by aspirin‐mediated acetylated COX‐2 (Figure 5) [10, 66].

According to evidence, cannabinoid receptor‐2 and the PPARs family receptors were found to act as the main receptors for PD1 and AT‐PD1 [67]. GPR37 also connects to PD1, and its interaction inhibits protein kinase A and induces calcium‐related signaling cascades, which lead to the induction of phagocytosis and regulate cytokine generation [68]. Eosinophils, airway epithelial cells, and other leukocytes, including macrophages and polymorphonuclear cells (PMNs), are the major sources producing PD1 [6, 69]. According to evidence, PD1 is also generated by human peripheral blood mononuclear cells in Th2‐type circumstances in an LOX‐dependent way through a 16 (17)‐epoxide intermediate. Moreover, PD1 functions to inhibit T cell migration in vivo, decrease TNF‐α and IFN‐γ generation, and boost T cell apoptosis [70]. PD1 has also been depicted to augment the phagocyte removal during acute inflammatory responses via regulating leukocyte influx, enhancing macrophage ingestion of apoptotic PMN neutrophils in vivo and in vitro [65]. Importantly, the production of PD1 has previously been found to be decreased in patients with severe asthma [71], unveiling the selective dysregulation in the enzymatic activity of 15‐LOX in these patients' eosinophils [44]. PD1 has moreover been illustrated to boost the resolution of the pulmonary inflammation and inhibit AHR. Intriguingly, PD1 acts to regulate the levels of IL‐13 and IL‐5 but not IL‐4 levels, proposing that ILC2 cells, rather than Th2 cells, are possibly the main cellular target for PD1 [72].

Plasma levels of PD1 and the ratio of PD1 to IL‐6 cytokine have recently been reported to display a gradual reduction across control, low‐risk, medium‐risk, and high‐risk groups of CHD and showed a negative association with the SYNTAX score. The correlations analysis also uncovered the negative associations between plasma PD1 and both inflammatory CRP and IL‐6 mediators, and positive associations with the anti‐inflammatory IL‐10 cytokine. Further, plasma PD1 has been demonstrated to provide the predictive value for short‐lasting prognosis, whereas the PD1 to IL‐6 ratio was considered to be indicative of long‐lasting prognosis [40].

In the context of atherosclerosis, PDs have received great interest owing to their potential role in modulating inflammatory processes and contributing to cardiovascular health [73]. The understanding of PDs in the context of atherosclerosis opens avenues for therapeutic interventions. Strategies aimed at enhancing the production of PDs or administering them directly could help mitigate the inflammatory processes and oxidative stress associated with atherosclerosis. Additionally, omega‐3 fatty acid supplementation may be a potential way to enhance the body's levels of PDs, promoting cardiovascular health. As our understanding of their mechanisms and effects deepens, they may offer novel targets for therapeutic strategies aimed at preventing and treating atherosclerotic cardiovascular diseases. Further studies are warranted to elucidate their full potential and practical applications in clinical settings.

#### 5.2.3 Macrophage Mediators in Resolving Inflammation (MaRs)

Among the known immunoresolvents, MaRs were initially figured out by Serhan and colleagues in 2009 and have shown broad protective functions in view of wound healing, tissue regeneration, resolving inflammation, and organ protection [74, 75, 76]. The term maresin originates from the phrase “macrophage mediators in resolving inflammation.” MaRs are constructed from the omega‐3 fatty acid DHA via a series of enzymatic reactions [6]. Currently, the MaRs family predominantly encompasses MaR1, MaR2, and MaR conjugate in tissue regeneration, and MaR‐like lipid mediators. They have different names based on the position and conformation of the double bond and hydroxyl group. As implied in their nomenclature “macrophage mediators in resolving inflammation,” macrophages were initially identified as the main source of MaRs, whereas platelets, neutrophils, and other cell kinds have been indicated to also be able to generate MaRs [77]. Tissue macrophages that express 12‐LOX can turn DHA into 13S, 14S epoxy‐MaR, which can subsequently be turned into MaR1 and MaR2 through enzymatic hydrolysis. Besides, the interaction that occurs between human platelets and neutrophils during vascular inflammation can turn DHA into 13S, 14S epoxy‐MaR via 12‐LOX expressed on platelets. More changes of the 13S, 14S epoxy‐MaR, which is mediated by neutrophils, can generate MaR1 (Figure 5) [10, 74, 78, 79].

MaR1 was initially recognized in human monocyte‐derived macrophage cultures. MaR1 increases macrophage‐mediated uptake and phagocytosis of human apoptotic neutrophils via efferocytosis. Likewise, MaR1 restricts the neutrophil influx into the inflamed peritoneum of mice [61, 80]. The reported receptors for MaR1 comprise BLT1 and the human leucine‐rich repeat‐containing G protein‐coupled receptor 6 (LGR6), which is a stereoselective MaR1 efferocytotic receptor. Furthermore, MaR1 may function as an endogenous agonist for the nuclear receptor retinoic acid‐related orphan receptor α, which is responsible for regulating the polarization of M2‐type macrophages and has anti‐inflammatory features [73, 81, 82]. Notably, M2‐type macrophages or alternatively activated macrophages have been indicated to exert a crucial role in inhibiting inflammation and endocytosing apoptotic cell debris [83]. In this regard, Dalli and colleagues' study revealed that MaR1 possesses the action in influencing the phenotype of macrophages, tending to polarize towards the M2‐type macrophages, enhancing efferocytosis, phagocytic capacity, and also enhancing tissue regeneration [79, 84]. According to evidence, MaR1 fulfills its bioaction through the interaction with the ALX/FPR2 in multiple model of inflammatory illness, and the beneficial impact of MaR1 could be blocked by using ALX/FPR2 antagonist, therefore suggesting that ALX/FPR2 might be additional GPCR for MaR1, even though the direct connecting of MaR1 to ALX/FPR2 has yet to be investigated [77, 85, 86]. Of note, the receptor for MaR2 has yet to be discovered and requires further investigations [77].

MaRs have been shown to exert a pivotal role in beginning and augmenting the pro‐resolving bioactions of phagocytes, reducing the severity of the overall inflammatory response and thus protecting against inflammation‐associated illnesses [77]. Also, MaR1 interaction with LGR6 through coupling a Gαs protein to induce cAMP leads to inducing efferocytosis, increasing phagocytosis, and diminishing the chemotaxis of monocytes/macrophages and polymorphonuclear leukocytes toward the site of inflammation [81]. In atherosclerosis, chronic inflammation drives plaque formation and progression. MaRs can help mitigate this inflammation by boosting the macrophage‐mediated clearance of apoptotic cells and cellular debris, thereby reducing the inflammatory response and preventing secondary necrosis. This action can contribute to plaque stability and prevent plaque rupture. Also, MaRs can help improve endothelial cell survival, function, and proliferation, thereby potentially reducing the risk of atherosclerotic lesion formation [77]. Growing evidence has illustrated that lymphocytes, especially T cells, exert a crucial role in atherosclerosis pathogenesis. Importantly, MaR1 has been known as a key modulator of acquired immunity because it inhibited the induction of effector T cells and augmented the induction of Treg cells, therefore reducing the generation of pro‐inflammatory cytokines and enhancing the generation of anti‐inflammatory cytokines [87]. Nonetheless, the precise function of MaR1 on lymphocytes and atherogenesis is far from understood and requires more research.

More recently, increased plasma concentrations of MaR1 were found to be associated with a lower risk of atherosclerotic cardiovascular disease development [88]. Besides that, the study by Yin et al. indicated that the serum concentrations of MaR1 are decreased in hypertensive patients and are negatively associated with systolic blood pressure. Also, intraperitoneal (I.P.) administration of MaR1 in the angiotensin II (AngII) infusion‐induced hypertension murine model diminished the enhancement of blood pressure and declined vascular remodeling. Moreover, the treatment of vascular smooth muscle cells (VSMCs) with MaR1 decreased extreme proliferation, phenotype switching, and migration, as well as dysregulated pyroptosis. Nonetheless, it was found that knocking out the MaR1 receptor, that is, LGR6, deteriorates the pathological vascular remodeling, which was not reversible with additional treatment with MaR1. Therefore, MaR1 functions to implicate the signaling pathway of the Ca^2+^/calmodulin‐dependent protein kinase II/nuclear factor erythroid 2‐related factor 2/heme oxygenase‐1, thereby regulating vascular remodeling via LGR6. In sum, the findings of this study indicated that MaR1 supplementation may become a new approach to therapy for preventing and treating hypertension [89]. In *ApoE^−/−^* mice, increased levels of LTB4 were reported to be directly correlated with plaque vulnerability, while MaR1 was associated with plaque stability, indicating the imbalance between pro‐inflammatory LTB4 and pro‐resolving MaR1 lipid mediators during atheroprogression [63]. Moreover, MaR1 has been shown to alleviate cardiac injury and protect against cardiac dysfunction and may be useful in decreasing sepsis‐induced cardiac injury [90]. Given the salutary roles of MaRs in resolving inflammation and tissue repair, they represent a promising therapeutic target for the treatment of atherosclerosis and its complications. Strategies that enhance the production of MaRs, such as diet (increased omega‐3 fatty acid intake) or the development of novel therapeutic agents based on MaR‐like structures, could be valuable in managing atherosclerotic disease. Therefore, MaRs might offer new insights into the development of therapeutic strategies aimed at treating atherosclerosis and reducing the risk of cardiovascular events [77, 91]. Ongoing research is necessary to fully understand their mechanisms of action and explore their potential as therapeutic agents in cardiovascular diseases. Tables 1 and 2 summarize the role and therapeutic potential of immunoresolvents in atherosclerosis, respectively.

**Table 1:** The role of immunoresolvents in atherosclerosis.

Mediator	Condition	Site	Expression changes	Role	References
ASA‐triggered lipoxin (ATL)	Symptomatic peripheral artery disease	Plasma	Decreased	Reduced ATL concentration is involved in atherosclerosis pathogenesis.	[1]
LXA4	Acute myocardial infarction	Plasma	Increased	High levels of plasma LXA4 are linked with a lower risk of recurrent ischemic events for patients with acute myocardial infarction.	[39]
LXA4	Coronary heart disease (CHD)	Plasma	Decreased	The ratio of LXA4 to IL‐6 is indicative of long‐lasting prognosis.	[40]
AT‐LXB4	Coronary artery disease (CAD)	Plasma	Decreased	Reduced plasma AT‐LXB4 levels are implicated in CAD pathogenesis.	[41]
RvE1	CAD	Plasma	Decreased	Decreased plasma RvE1 levels are implicated in CAD pathogenesis.	[41]
RvE1	Atherosclerosis	Serum	Increased	Imbalanced RvE1 and LTB4 might contribute to failing vascular inflammation resolution and subsequent advancement toward chronic inflammation in atherosclerosis.	[4]
RvD1	Carotid endarterectomy	Vulnerable human plaques	Decreased	Decreased RvD1 is involved in carotid endarterectomy.	[5]
RvD1	CHD	Plasma	Decreased	The ratio of RvD1 to IL‐6 is indicative of long‐lasting prognosis.	[40]
RvD1, RvD2, RvD3, RvD5, and AT‐RvD3	CAD	Plasma	Decreased	Decreased plasma levels of RvD1, RvD2, RvD3, RvD5, and AT‐RvD3 may contribute to the CAD pathogenesis.	[41]
PD1	CHD	Plasma	Decreased	Plasma PD1 provides the predictive value for short‐lasting prognosis, whereas the PD1 to IL‐6 ratio is indicative of long‐lasting prognosis.	[40]
MaR1	Atherosclerotic cardiovascular disease (ASCVD)	Plasma	Increased	Increased plasma MaR1 levels are associated with a lower risk of ASCVD development.	[88]
MaR1	Hypertension	Serum	Decreased	Serum MaR1 concentrations were negatively associated with systolic blood pressure in hypertensive patients.	[89]

**Table 2:** Therapeutic effects of immunoresolvents in atherosclerosis.

Mediator	Model type	Administration mode	Effects	References
ATL	Human saphenous vein smooth muscle cells (HSVSMCs)	Co‐culture	ATL blocks platelet‐derived growth factor (PDGF)‐stimulated migration of HSVSMCs and reduces the phosphorylation of the platelet‐derived growth factor receptor‐β (PDGFR‐β).	[1]
ATL	Apolipoprotein E‐deficient (*ApoE* ^−^^/^^−^) mice	Osmotic pumps loaded with ATL	ATL blocks atherosclerosis advancement in the aortic root and thoracic aorta of *ApoE* ^−/−^ mice. Likewise, ATL decreases the influx of macrophages and apoptotic cells in atherosclerotic lesions.	[38]
RvE1	HSVSMCs	Co‐culture	RvE1 blocks PDGF‐stimulated migration of HSVSMCs and diminishes the phosphorylation of the PDGFR‐β.	[1]
RvE1	Atherosclerosis rabbit model	Oral/topical	RvE1 considerably decreased atherogenesis and prevented periodontitis. In the absence of periodontitis, oral/topical application of RvE1 led to dramatically less arterial plaque, a lower intima/media ratio, and reduced inflammatory cell influx relative to no treatment. Local oral RvE1 application also markedly diminished systemic C‐reactive protein (CRP) levels.	[54]
RvE1	An experimental model of diet‐induced atherosclerosis, that is, the ApoE*3Leiden transgenic murine	Oral	RvE1 attenuates atherosclerotic lesion development in mice with established progressive atherosclerosis. Also, RvE1 decreases total lesion area by 35%, and in animals treated with RvE1, the proportion of atherosclerotic lesions considerably skews towards mild (type I) lesions over more severe (type IV and V) lesions.	[55]
RvD1	Fat‐fed *Ldlr* ^−^^/^^−^ mice	I.P.	Injecting RvD1 into these mice during plaque progression restores the RvD1:LTB4 ratio to that of less advanced lesions and augments plaque stability, comprising reduced lesional oxidative stress and necrosis, improved lesional efferocytosis, and thicker fibrous caps.	[5]
MaR1	AngII infusion‐induced hypertension, murine model	I.P.	MaR1 treatment diminishes the enhancement of blood pressure and declines vascular remodeling.	[89]

Abbreviations: AngII, angiotensin II; ASA, acetylsalicylic acid; I.P, intraperitoneal.

## 6 Obesity‐Driven Inflammation, Depot Biology, and Dysregulated Resolution

Obesity‐driven inflammation is generally characterized as a chronic, low‐grade inflammatory state that underlies many cardiometabolic complications. In obesity, excessive nutrient intake leads to adipocyte hypertrophy and hyperplasia, causing disturbance of tissue homeostasis. Enlarged adipocytes experience hypoxia owing to insufficient vascularization, triggering cellular stress responses and promoting the release of chemokines such as monocyte chemoattractant protein‐1 (MCP‐1/CCL2). These signals recruit circulating monocytes into adipose tissue, where they differentiate into macrophages. Importantly, the macrophage population shifts from an anti‐inflammatory M2‐like phenotype toward a pro‐inflammatory M1‐like phenotype. This polarization results in increased secretion of cytokines, including TNF‐α, IL‐6, and IL‐1β, which activate intracellular signaling pathways such as NF‐κB and c‐Jun N‐terminal kinase, amplifying inflammatory gene expression. In addition, inflammasome activation, especially NOD‐like receptor protein 3, further enhances IL‐1β generation. The chronic presence of these inflammatory signals disrupts insulin signaling pathways, contributing to systemic insulin resistance. Endothelial cells are also affected, leading to vascular dysfunction and accelerated atherogenesis. Thus, adipose tissue in obesity becomes an active immunometabolic organ that sustains systemic inflammation rather than serving merely as an energy reservoir. Notably, obesity can also be conceptualized not only as a pro‐inflammatory condition but also as a resolution‐defective state [92, 93].

### 6.1 Obesity as a Resolution‐Defective State Sustaining Vascular Inflammation

Atherosclerosis is increasingly recognized not merely as a lipid‐storage disorder but as a chronic failure of inflammatory resolution within the cardiometabolic axis. In this context, obesity represents a prototypical “resolution‐defective” state, characterized not only by persistent activation of innate immune pathways but also by impaired engagement of endogenous pro‐resolving circuits. Rather than resolving after nutrient excess subsides, adipose inflammation becomes self‐sustaining through defective efferocytosis, reduced immunoresolvent biosynthesis, impaired macrophage phenotypic switching, and persistent inflammasome activation. This defective resolution state promotes sustained cytokine spillover, endothelial activation, and maladaptive vascular remodeling, key drivers of atherogenesis and plaque vulnerability. Importantly, resolution failure in obesity is not simply systemic but depot‐specific, with visceral and cardio‐proximal depots exhibiting heightened susceptibility to chronic inflammatory imprinting [92].

### 6.2 Depot‐Specific Immunometabolic Signaling and Cardio‐Proximal Crosstalk

Adipose tissue heterogeneity is central to understanding obesity‐driven vascular inflammation. Visceral adipose tissue, epicardial adipose tissue (EAT), and perivascular adipose tissue (PVAT) display distinct immunometabolic programs that directly affect adjacent vascular beds. Cardio‐proximal depots, like EAT and PVAT, are uniquely positioned to modulate vascular biology through paracrine and vasocrine signaling. Unlike subcutaneous fat, these depots lack fascial barriers and share microcirculatory continuity with coronary arteries and myocardium. In obesity, they undergo qualitative remodeling characterized by enrichment of pro‐inflammatory macrophage subsets, increased IL‐1β, TNF‐α, and MCP‐1 production, reduced adiponectin and apelin signaling, oxidative stress and endothelial uncoupling, and impaired immunoresolvents biosynthetic capacity [92].

### 6.3 Persistent “Adipose Memory” and Chronic Unresolved Vascular Inflammation

Emerging multi‐omic evidence indicates that obesity imprints durable transcriptional and epigenetic changes across adipocytes, stromal cells, and immune compartments. Even after significant weight loss, depot‐specific inflammatory and fibrotic programs remain only partially reversible. This phenomenon, termed “adipose memory,” is characterized by persistent enhancer/promoter accessibility at inflammatory loci, sustained macrophage polarization toward lipid‐associated phenotypes, transforming growth factor β‐driven extracellular matrix remodeling, impaired pro‐resolving mediator biosynthesis, and exaggerated inflammatory reactivation upon metabolic re‐challenge. In cardio‐proximal depots, like EAT, immune‐memory phenotypes, including tissue‐resident macrophages and memory T cell subsets, may persist despite normalization of body weight. This provides a plausible mechanistic bridge between prior obesity exposure and continued coronary inflammation. The study of Karakasis et al. frames this as a failure of resolution biology rather than incomplete metabolic correction. In this model, adipose depots that have once transitioned into a pro‐inflammatory state do not automatically revert to immunometabolic quiescence, thereby sustaining vascular inflammatory tone even after systemic metabolic improvement [92].

### 6.4 Translational Implications–Resolution Pharmacology in Cardiometabolic Disease

The recognition of obesity as a resolution‐defective and depot‐imprinted inflammatory condition has direct therapeutic implications. Traditional anti‐inflammatory strategies targeting upstream cytokines (e.g., IL‐1β, TNF) may attenuate inflammatory amplitude but do not necessarily restore resolution circuitry. Moreover, systemic cytokine blockade may carry immunosuppressive risks. Resolution pharmacology, leveraging immunoresolvent analogs or receptor‐targeted agonists, aims to enhance efferocytosis within adipose and vascular tissues, reprogram macrophage phenotypes toward reparative states, restore endothelial integrity, normalize adipokine flux from cardio‐proximal depots, and interrupt adipose–vascular inflammatory feed‐forward loops. Nonetheless, several translational challenges remain that include pharmacokinetic instability of native immunoresolvents, limited clinical biomarkers of target engagement, depot‐specific delivery considerations, and incomplete understanding of long‐lasting immune reprogramming effects. Thus, while immunoresolvent‐based interventions are biologically plausible and mechanistically aligned with the concept of resolution restoration, careful clinical validation in cardiometabolic populations is required [92].

## 7 Pharmacokinetics and Therapeutic Development Challenges

Although dietary EPA/DHA supplementation increases precursor availability, endogenous immunoresolvent generation depends on enzymatic context and inflammatory status, and circulating immunoresolvent levels following supplementation are typically low and variable [94]. Thus, omega‐3 intake represents substrate enrichment rather than resolution pharmacotherapy.

While many preclinical studies report favorable effects of immunoresolvents on plaque stability and inflammation resolution, most data derive from murine *ApoE^−/−^* or *LDLR^−/−^* models that incompletely recapitulate human plaque biology. Human studies remain limited in size and are largely cross‐sectional, precluding causal inference. Notably, reduced circulating immunoresolvent levels in patients with cardiovascular disease may reflect not only impaired biosynthesis but also increased local consumption, compartmentalization within plaques, altered enzymatic activity (e.g., 5‐LOX, 15‐LOX), or accelerated metabolic inactivation. Thus, decreased plasma immunoresolvent concentrations cannot be interpreted uncritically as causative. Native immunoresolvents exhibit significant pharmacokinetic liabilities, including rapid enzymatic degradation, short half‐lives, limited oral bioavailability, and susceptibility to oxidation [8]. These properties complicate systemic administration and necessitate exploration of stabilized analogs, receptor agonists, or targeted delivery systems such as nanoparticle‐based carriers. Safety, optimal dosing, receptor selectivity, and potential off‐target GPCR interactions are also discussed. Importantly, large‐scale clinical trials of direct immunoresolvent administration in atherosclerosis are currently lacking, highlighting a major translational gap. In contrast to current therapies such as IL‐1β inhibitors, which suppress inflammatory pathways, immunoresolvents aim to restore physiological resolution rather than block inflammation. However, unlike these agents, immunoresolvent‐based therapies have not yet demonstrated clinical efficacy in large randomized trials. Major barriers include instability, difficulties in achieving sustained therapeutic concentrations, lack of standardized biomarkers of target engagement, and incomplete understanding of dose–response relationships [94]. To overcome instability and rapid metabolism, synthetic stable analogs of LXs and Rvs, as well as small‐molecule receptor agonists targeting ALX/FPR2 or ChemR23, have been developed. These compounds aim to preserve pro‐resolving signaling while improving pharmacokinetic properties and receptor selectivity. Preclinical studies suggest that structurally modified analogs resist metabolic degradation and maintain anti‐inflammatory and pro‐resolving bioactivity [95, 96]. Nevertheless, receptor pleiotropy and ligand bias introduce translational complexity. For example, ALX/FPR2 can generate pro‐resolving or pro‐inflammatory outputs depending on ligand context. Therefore, therapeutic agonists must demonstrate signaling bias favoring resolution pathways without unintended activation of inflammatory cascades. This necessitates rigorous pharmacodynamic characterization [35, 97]. Additionally, given the short half‐life and potential off‐target distribution of lipid mediators, targeted delivery systems, such as nanoparticle encapsulation, liposomes, or lesion‐targeted carriers, are being explored to enhance tissue‐specific accumulation and reduce systemic exposure. Preclinical studies indicate that nanoparticle‐mediated delivery of pro‐resolving mediators can improve plaque targeting and therapeutic efficacy in atherosclerosis models [5, 98]. However, performing further studies is warranted in this area.

## 8 Conclusions and Future Directions

Atherosclerosis, a chronic inflammatory disease characterized by the buildup of plaque within the arterial walls, is closely associated with persistent and chronic unresolved inflammation. Immunoresolvents facilitate the resolution phase of inflammation by promoting the effective clearance of apoptotic cells and debris (i.e., efferocytosis) and restraining the recruitment of pro‐inflammatory leukocytes to the loci of inflammation, indicative of their dual feature of pro‐resolution and anti‐inflammation. Immunoresolvents can influence the activity of various immune cells, including macrophages, neutrophils, and T‐cells. They induce macrophages to adopt an anti‐inflammatory M2‐type phenotype and to downregulate pro‐inflammatory mediators like LTB4. These mediators can boost the timely resolution of inflammation without causing immunosuppression. The resolution process is crucial for stabilizing atherosclerotic plaques and preventing complications like plaque rupture [99]. By promoting a balanced endothelial function, immunoresolvents can improve vasodilatory responses and reduce the expression of adhesion molecules, thereby decreasing leukocyte recruitment to the arterial wall. Immunoresolvents also play a role in altering lipid metabolism, potentially reducing lipid accumulation within plaques. As discussed in this mini‐review, recent evidence shows that imbalanced pro‐inflammatory lipid mediators and immunoresolvents may contribute to the pathogenesis of atherosclerosis. Besides, resolving inflammation is the most significant physiological mechanism that is dysregulated in atherosclerosis [100]. Also, impaired activity of the key enzymes involved in the biosynthesis of immunoresolvents, such as 15‐LOX, is thought to potentially contribute to the diminished production of immunoresolvents, dysregulation of resolving inflammation, and consequently the pathogenesis of some diseases. Research is ongoing to develop therapies that can enhance the generation or signaling of immunoresolvents. This could include supplements rich in omega‐3 fatty acids (e.g., fish oil) or synthetic analogs of existing immunoresolvents. Deciphering the individual variations in immune responses and lipid metabolism, as well as receptor pleiotropy and ligand bias of some immunoresolvents, is of high importance and potentially requires further investigations.

In conclusion, immunoresolvents play a significant role in modulating the immune response in atherosclerosis, presenting exciting biological insights and therapeutic opportunities. Ongoing research will be crucial to fully exploit their potential in cardiovascular disease prevention and management.

## Author Contributions

**Ramin Lotfi:** conceptualization, methodology, validation, writing – original draft, writing – review and editing. **Alireza Nasirifar:** investigation, writing – original draft, writing – review and editing, methodology, visualization. **Hoda Rahmani:** investigation, writing – original draft, writing – review and editing. **Sara Falahi:** investigation, writing – original draft, writing – review and editing. **Meysam Kashiri:** investigation, writing – original draft, writing – review and editing. All authors listed have made a substantial, direct, and intellectual contribution to the study and approved it to be published.

## Funding

The authors have nothing to report.

## Conflicts of Interest

The authors declare no conflicts of interest.

## Transparency Statement

The corresponding author, Ramin Lotfi, affirms that this manuscript is an honest, accurate, and transparent account of the study being reported; that no important aspects of the study have been omitted; and that any discrepancies from the study as planned (and, if relevant, registered) have been explained.

## Data Availability

Data sharing does not apply to this article as no datasets were generated or analyzed during the current study. The sources of data utilized for the preparation of the manuscript have been mentioned in the references. No new data was created or analyzed in this research.
